# Science in the time of COVID-19: Reflections on the UK Events Research Programme

**DOI:** 10.1038/s41467-022-32366-1

**Published:** 2022-08-10

**Authors:** Theresa M. Marteau, Michael J. Parker, W. John Edmunds

**Affiliations:** 1 University of Cambridge, Department of Public Health and Primary Care, Cambridge, UK; 2 University of Oxford, Nuffield Department of Population Health, Oxford, UK; 3 London School of Hygiene and Tropical Medicine, Department of Infectious Disease Epidemiology, London, UK

**Keywords:** Viral infection, Scientific community, Epidemiology, SARS-CoV-2

## Abstract

We reflect on the extent to which the UK Events Research Programme adhered to four principles of design and evaluation in assessing risk of transmission from attending such mass events as football matches and festivals, and lessons learned.

Attending mass gatherings such as festivals, plays or football matches carries some increased risk of transmission of SARS-CoV-2. But how much? And how effective are different measures at reducing this risk? The UK Government’s *Roadmap out of lockdown*^1^ published in February 2021, included a commitment to set up a series of studies to guide decisions about the safe re-opening of these crucial elements of our cultural and economic lives. *The Events Research Programme* (ERP) was launched shortly afterwards^2^. Given legal restrictions on public gatherings at this time, ministerial derogation was required for events to occur. Events had to contribute to scientific research. The ERP governance structure included a Science Board of which all authors were members, chaired by TMM^2^. The Science Board in turn was guided by a Science Framework prepared by a working group – of which we were members - organised by a subgroup of the Scientific Advisory Group for Emergencies (SAGE). This set out two priority research questions for the ERP concerning risk of transmission and four principles to guide the design and evaluation of studies addressing these^3^ [Box 1].

So, how well did the ERP address the two priority research questions and adhere to the four guiding principles of design and evaluation? What impact has it had on policy? And what lessons can we learn for future policy-relevant evaluations?

First and foremost, the ERP did deliver the UK government’s commitment to a research programme to inform the safe re-opening of events. The ERP put in place a series of studies to estimate the risk of transmission of SARS-CoV-2 associated with attendance at different types of events and how to mitigate these risks whilst allowing ticketed commercial events to take place. The scale, scope and speed of the Programme is worth emphasising. It took place in three phases over a three-month period (17 April to 25 July 2021, Fig. 1), with studies undertaken at 120 events across 28 different venues in England with around 2 m attendees^2^. Events took place in a range of settings and venues—indoor and outdoor, small and large, seated and unstructured - involving passive and active audiences, ranging in length from a few hours to a few weeks. Events in the devolved nations were not set up given the short timeframe. Study methods included testing of participants and linking to wider public health datasets, detailed monitoring of ventilation, video capture and analysis of crowd behaviour, interviews and surveys. The early events (Phase I) operated at a lower capacity, largely with physical distancing and rigorous testing before entry. Phase II events were larger, operating at near full capacity, though testing and other measures remained in place. Phase III events were conducted close to pre-pandemic conditions.

**Fig. 1:**
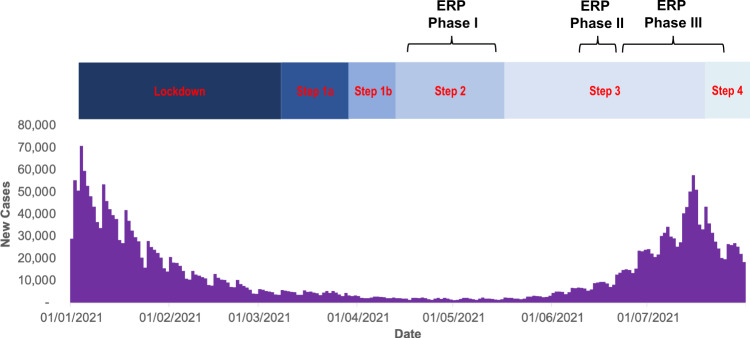
Timelines for the three phases of the Events Research Programme (ERP), steps out of lockdown, and new cases of COVID-19 in England. Details of steps out of lockdown can be found here^1^. NB: Step 4 was delayed until 19th July. Details of the ERP events are given here^2,6,17–19^. Data on the new confirmed cases of COVID-19 in England were taken from the UK Government COVID-19 dashboard^20^.

The ERP involved collaboration and coordination between a diverse range of academic and consultancy teams, several government departments, national and local public health teams, event organisers and the public. Successfully carrying out research across all these events within a matter of weeks was a huge undertaking and an unparalleled achievement. Inevitably, given the scale and complexity of a programme to be delivered at speed, not all challenges were overcome.

## Priority questions

The two priority questions set out in the Science Framework were addressed as summarised in Box 1. The findings—yet to be reported in full in peer-reviewed publications—significantly improved our understanding of the risks of COVID-19 associated with attendance at events and how to mitigate the spread of the virus. Greater adherence to the four principles of design and evaluation would, however, have strengthened the resulting evidence.

Box 1 Principles for design and evaluation, priority questions^3^ and summary findings^6,19^

I. Principles for design and evaluation
  i. Design: a range of studies and study designs is needed to optimise causal inference ranging from randomisation for large-scale events to meta-analyses of case-control and cohort studies for smaller events designed to allow comparison.
  ii. Measures: core set of measures across studies—biological; environmental; behavioural
  iii. Ethical: generate high-quality evidence, transparently, treating everyone with equal moral value, with minimal suffering
  iv. Open science: use the practices of open science, including pre-registration of protocols
II. Priority research questions
  i. Given a pre-specified set of mitigation measures, is there evidence of an increased risk of transmission of SARS-CoV-2 from attendance at (a) outdoor and (b) indoor events?
  **Phase I**
    Based on pre-post-event testing:
    28 PCR positive cases were detected in Phase I, with 11 assessed to be positive before the event (despite the requirement for a negative Lateral Flow test for admittance) and 17 assessed to be infected at or after the event.
      ° It is very difficult to make any inference about the risk of transmission from these figures, given the very low return rate of pre- and post-event PCR tests (only 15% returned both tests), the low prevalence at the time of the studies and the lack of a comparator group for the studies.
  **Phase III**
    Based on a self-controlled case-series study which analysed data on 3357 PCR positive cases, 1149 of these tested positive during the base-line periods (days 1–2 and 10–16 after the event), and 2208 during the putative risk period (days 3-9 after the event):
    Attendance at mainly outdoors unseated events (mostly music festivals) was associated with an increased risk of acquiring SARS-CoV-2 (rate ratio 1.70, 95% CI 1.52–1.89).
    Attendance at other events (including theatre and sporting events) was not associated with a significantly increased risk of infection.

ii. Which characteristics of events and venues and behaviours likely contribute most to transmission?
  Uncertainty remains about factors that likely contributed to the increased risk of transmission observed at music festivals in Phase III, including behaviour at the event, event size and duration, mode of travel to and from the event, age distribution and vaccination coverage of attendees.
    Based on CO_2_ monitoring in 179 different spaces across 55 events in Phases I, II & III
  Maximum recorded levels were <1500 parts per million in 161/179 spaces monitored; average CO_2_ levels were <800 ppm in 170/179 spaces, suggesting they were generally well ventilated.
    Based on behavioural monitoring at over 21 events with over 9300 h of video footage:
  Unstructured settings were more likely to be associated with pockets of high crowdings, such as at entrances, exits, refreshment facilities and toilets.
  35% of attendees across Phases I, II and III events wore face coverings, 49% when they were required and 18% when they were not.

## Adherence to design and evaluation principles

### Design

A range of study designs was identified in the Science Framework^3^ to optimise the ability to infer causality between events, mitigations and risk of transmission, including randomised controlled studies and meta-analyses across events. Despite drafting a protocol with sample size calculations for a large randomised controlled trial^3^, no such design was adopted, possibly due to perceived operational difficulties. The lack of suitable control groups, combined with low prevalence during the initial phases of the ERP (Fig. 1), relatively small crowds and the low rate of PCR testing (see later) made it difficult to estimate the risk of transmission during Phases I and II. The larger sizes of Phase III events combined with higher prevalence at that time and the adoption of the self-controlled case-series analysis^4^ provided the potential to draw stronger conclusions about transmission risk.

The programme attempted to cover a wide range of events and venues. The selection of events involved many different parties (the Science Board, public health bodies, local and central government and event organisers) and so did not just reflect scientific needs. Transparent, mainly design-based criteria for choosing events would have strengthened the programme and increased levels of understanding among attendees. It might also have increased confidence amongst the wider public and others in the scientific integrity of the ERP^5^.

### Core measures

To facilitate the pooling of data across studies, the ERP aimed to collect a common set of standardised measures. The risk of transmission proved the most difficult. In Phases I and II, the risk of transmission was assessed by requiring participants to undertake two PCR tests, one within 48 h prior to attending and another 5 days after the event. A separate, recent (ideally 24 h before the event but sometimes up to 72 h) negative lateral flow device (LFD) test was required to gain entrance to the venue or a certificate of vaccination (Phases II and III). These tests had different purposes. The LFD was required to reduce the risk of transmission at events. The PCR test was for research purposes. However, this distinction was frequently lost on attendees and organisers, likely contributing to low rates of return of PCR tests, estimated at 15% (7764/51,319) across events in Phase I, ranging from 3 to 61%^6^. This reflected several problems. First, poorly integrated testing and ticketing systems (e.g. the need to order each of three required tests separately through the national system). Second, ineffective communication of requirements for participation in the ERP. While tickets were only issued in Phase I events to those who had signed a consent form, there was insufficient time to evaluate how well those signing the consent form understood what was required of them. The Science Board requested that incentives were offered for test returns, given higher PCR return rates observed in Phase I when these were offered^6^. There was, however, insufficient time to resolve what transpired to be unfounded legal concerns about the use of incentives.

These problems in assessing the risk of transmission using multiple PCR tests in all participants were circumvented by the adoption of a self-controlled case-series design in Phase III^4^. This design could assess the risk of transmission from attending events without requiring pre and post-event testing in all participants. The method relied solely on routine PCR testing run through the national surveillance and contact tracing system, NHS Test and Trace. Ethics approval was granted on the understanding that while written consent was not required, event organisers would ensure that all participants received written information that attendance was conditional upon participating in the ERP. For one event—a European football tournament—many tickets were sold prior to the ERP, and the event organisers were unwilling or unable to inform ticket holders that their attendance was conditional upon participation in the study. For two other events in Phase III, written consent was sought by the event organisers despite it not being a condition of ethics approval. For one event, fewer than 5% signed consent forms which blocked receipt of data from 95% of over 300,000 attendees at the multi-day event. For the other event, also multi-day, 17% of around 350,000 attendees signed consent forms, again blocking receipt of their data. The study power was significantly reduced by these restrictions and other - as yet - unfathomed reasons preventing access to data for some of the most well-attended events.

The need to share and link data—with appropriate regulatory oversight—is one of the ten lessons from the pandemic highlighted in a report from the Office for Statistical Regulation (OSR)^7^.

### Ethics

Ethical considerations guiding the Science Board drew upon the report of an international working group, chaired by one of us (MP), on conducting research in global health emergencies^8^. This highlighted the importance in such contexts of generating high-quality evidence to contribute to reducing harm, treating everyone involved with equal moral respect, and effective engagement.

The Science Board’s strong advice was that the event organiser should obtain written, informed consent from participants to provide some reassurance that they understood that events were part of the ERP, attending likely involved some additional risk over non-attendance and that the success of the ERP in generating useful knowledge required that they undertake pre- and post-event tests (in Phases I and II) and return the results to the programme. Written consent was required as part of research ethics committee approvals for studies of the risk of transmission in Phases I and II but not for Phase III, for which event organisers were required only to inform ticket holders that attendance involved research participation. Failure to meet this requirement in practice resulted in a loss of access to much data, as described above in Core Measures. Partly in response to this, at the Science Board’s recommendation, a Data Monitoring and Ethics Committee was set up In Phase III, independent of the Science Board, to receive and investigate any safety concerns about events included in the ERP^9^.

### Open science

Open Science principles^10^—the norm for most health and medical research—are not yet the norm for policy evaluation. Making explicit these principles to guide the work of the ERP was, therefore, unusual but consistent with the use of open science principles advocated by SAGE^11^, with the potential to strengthen the evidence generated. They were not always adhered to. While protocols for all studies were published before data collection was completed, only some of those in Phase I were published before data collection had started. In part, this reflected the extremely challenging timelines that research teams were working to, with data collection starting before some protocols were finalised. Once completed, publication of Phase I findings was delayed by being processed as a policy document—requiring ministerial approval for publication—rather than a scientific document. Clarification from the Government Office of Science removed this obstacle to the timely publication of Phase II and III results.

Against the standards set by the Code of Practice for Statistics by the Office for Statistical Regulation (OSR), data were, on occasion, released early without accompanying information to allow their quality, including their interpretation, to be judged. More broadly, Open Science is about informing the public of what is being done on their behalf as a basis for them to judge its trustworthiness, core to fostering trust^12^. Following the ten lessons from the pandemic set out by the OSR will avoid this and should promote the appropriate use of statistics for the public good in a way that promotes public confidence^7^.

## Short-term impact of ERP on policy

What impact has the Events Research Programme had on policies for opening events? At the time when most restrictions were lifted in England on 19 July 2021, findings from Phase I were available^6^. Nightclubs, sporting matches and festivals resumed at full capacity in July 2021 with no discernible impact of the evidence available at that time to mitigate the risk of transmission. There were no legal requirements for attendees to show evidence of either vaccinations or a recent negative test for infection, or to wear face coverings. Nor were there requirements for organisers to monitor CO_2_ levels and act should levels be deemed too high, although some event organisers did adopt some of these measures on a voluntary basis. As the ERP results are published and disseminated more widely, it is hoped that they will inform policy and practice within and beyond the UK.

## Lessons

We take three key lessons from the ERP concerning optimising study designs, optimising access to existing datasets, and conducting evaluations as a default.

### Optimise study designs

There is a trade-off between acting immediately to meet policy demands with some compromises on study design *versus* having a short delay to strengthen the study. This difficult balance merits more scrutiny and—where justified—more push-back from the scientists working under time pressure to set up evaluations. The Science Framework^3^ provided a critical benchmark against which the strengths and limitations of the studies that proved possible were assessed and published as statements from the Science Board in advance of studies being analysed^13^.

### Optimise access to existing datasets

The ERP was compromised in its ability to generate a more reliable estimate of the risk of transmission by difficulties in obtaining permissions to access existing datasets from multiple agencies with different governance arrangements. The need to set up more efficient, protected systems for sharing and linking data is recognised by the Office for Statistical Regulation which notes the lifesaving impacts of such sharing and linkage, stating: This must be prioritised by governments beyond the pandemic^7^.

### Evaluate policies as robustly as possible as a default

Despite many challenges and compromises, the ERP was one of the largest non-drug science-based programmes informing UK COVID-19 policy. It also provides a model for generating evidence to inform policy that could and should be developed and applied both in and outside of emergencies. It comprised a co-ordinated, science-led research programme that had adequate resources and political support to achieve its aims. It has generated the most robust evidence to date on the risk of transmission at large events and the effect of mitigations. In doing so, the ERP is an exemplar of putting impact—as distinct from the process—evaluations at the heart of policy, with evaluations incorporated from the start, as recommended by the Office for Statistical Regulation and the Royal Statistical Society^7,14^. This is in stark contrast to the UK COVID-19 Test and Trace programme, which did not incorporate an impact evaluation. Instead, most evaluations were focused on process, conducted piecemeal by research consultants. This resulted in a dearth of high-quality evidence to realise the huge budget of £37bn for this programme^15^. Such robust evaluations were also missing for 92% of the UK government spend of £432bn on major projects in the year before the pandemic^16^. The absence of robust evaluations of major government projects is a norm that needs to change.

## Concluding comment

The lessons we have learned from our roles on the Science Board for ERP, together with the 20 lessons from the Office for Statistics Regulation and the Royal Statistical Society^7,14^, provide a sound basis for scientists, ethicists and policymakers to work together to generate systems and procedures to achieve robust evaluations of interventions as a default, resulting in better policy making both in and outside of emergencies. Despite its difficulties, the greatest mistake, both scientifically and ethically, would have been not to have set up the Events Research Programme.
